# Conventional cardiac resynchronization therapy upgrading and its effect on functional mitral regurgitation in patients with pacing-induced cardiomyopathy

**DOI:** 10.3389/fcvm.2026.1758374

**Published:** 2026-05-19

**Authors:** Agne Adukauskaite, Dalia Adukauskiene, Thomas Senoner, Valentin Bilgeri, Philipp Spitaler, Andrea Rubatscher, Wilfried Schgör, Markus Stühlinger, Bernhard Erich Pfeifer, Florian Hintringer, Wolfgang Dichtl, Fabian Barbieri

**Affiliations:** 1 University Hospital for Internal Medicine III (Cardiology and Angiology), Medical University Innsbruck, Innsbruck, Austria; 2 University Hospital of Lithuanian Health Sciences University, Kaunas, Lithuania; 3 Institute of Clinical Epidemiology, Tirol Kliniken, Innsbruck, Austria; 4 Institute of Electrical and Biomedical Engineering, University for Health Sciences, Medical Informatics and Technology (UMIT), Hall in Tirol, Austria; 5 Department of Cardiology, Angiology and Intensive Care Medicine, Deutsches Herzzentrum der Charité, Campus Benjamin Franklin, Berlin, Germany; 6 Department of Cardiology, Angiology and Intensive Care Medicine, Charité – Universitätsmedizin Berlin, corporate member of Freie Universität Berlin and Humboldt-Universität zu Berlin, Berlin, Germany; 7 Institute of Active Polymers and Berlin-Brandenburg Center for Regenerative Therapies, Helmholtz-Zentrum Hereon, Teltow, Germany

**Keywords:** cardiac resynchronization therapy, functional mitral regurgitation, mitral regurgitation, pacing-induced cardiomyopathy, secondary mitral regurgitation, upgrading

## Abstract

**Background:**

Data on the effects of conventional cardiac resynchronization therapy (CRT) upgrading by implantation of a left ventricular lead via the coronary sinus on functional mitral regurgitation (MR) in patients with pacing-induced cardiomyopathy are scarce.

**Methods:**

This analysis of the UPGRADE study, an investigator-initiated randomized controlled trial to evaluate the effect of conventional CRT on central sleep apnea, evaluated the significance of CRT on MR in all included patients. Differences in all-cause mortality were assessed in patients with significant MR during long-term follow-up.

**Results:**

Overall, 54 patients were included in the trial. MR was found in the majority of patients [mild: *n* = 30 (55.6%), moderate: *n* = 15 (27.8%), severe: *n* = 6 (11.1%)], while “none/trace” MR was observed in only three (5.6%) patients. CRT was associated with a significant reduction in MR [“none/trace”: *n* = 5 (9.6%), mild: *n* = 37 (71.2%), moderate: *n* = 6 (11.5%), severe: *n* = 4 (7.7%); *p* = 0.035)] after 3–5 months, with all patients either having an improvement in MR grading or it remaining unchanged. Notably, only two patients with severe MR improved to either a mild or moderate level. CRT upgrading was linked to a significant amelioration of vena contracta width [2 mm (1.0–4.3) at baseline versus 2 mm (1.0–3.0) post-CRT; r = 0.73, 95% CI 0.53–0.85, *p* < 0.001]. The presence of significant MR at baseline was associated with impaired survival during long-term follow-up [MR < 2: *n* = 14 (42.4%), MR ≥ 2: *n* = 19 (90.5%); hazard ratio 0.25 (95% CI 0.12–0.54); log rank *p* < 0.001].

**Conclusion:**

Conventional CRT upgrading in patients with pacing-induced cardiomyopathy was associated with significant improvement in MR grading. The presence of moderate or severe MR at baseline was associated with impaired outcomes during long-term follow-up, suggesting that these patients represent a high-risk cohort.

## Introduction

Pacing-induced cardiomyopathy (PICM) occurs in approximately 10%–20% of patients with permanent right ventricular (RV) pacing, leading to worse cardiovascular outcomes (1). Although different definitions have been proposed, PICM is usually defined as a reduction in left ventricular ejection fraction (LVEF) of <50%, with a decrease of ≥10% from initial LVEF in the case of permanent RV pacing, without finding another plausible cause for the LVEF deterioration, while symptoms or other signs of heart failure do not necessarily have to be present. This makes PICM an exclusion diagnosis (2). Some of the predisposing risk factors for developing PICM are male sex (2), higher RV pacing percentage with cutoffs varying from >20% to >60% (3–6), and longer duration of paced QRS suggesting greater dyssynchrony (2, 4, 5, 7). It has been postulated that RV pacing causes mechanical dyssynchrony, leading to pathological ventricular remodeling. It remains unclear, however, why the majority of patients with permanent RV pacing do not develop PICM, which suggests an as-yet unknown underlying individual susceptibility (8).

Upgrading a conventional one- or two-chamber pacing system to a cardiac resynchronization therapy (CRT) device has been shown to be an effective treatment for PICM. Introducing a left ventricular lead via the coronary sinus helps to achieve a better electrical and mechanical right/left-sided pacing synchrony and thus is able to reduce left ventricular end-diastolic volume, improve LVEF, and reduce heart failure symptoms and cardiovascular mortality in the majority of patients (9). Alternatively, upgrading to conduction system pacing (CSP), and thus causing more physiological electrical RV activation, can also improve LVEF in PICM (10). Echocardiographic detection of moderate or severe mitral regurgitation (MR) in the presence of RV pacing has been observed in approximately 29% of patients with normal LVEF who receive conventional pacemakers and was independently associated with worse cardiovascular outcomes (11). The prevalence of severe functional MR in the PICM population is yet to be reported.

It has been demonstrated that *de novo* CRT implantation can reduce functional MR in patients with heart failure and thus improve outcomes, underlining that it is an important and established therapy in treating heart failure patients with severe LVEF reduction (12). However, it remains to be shown how conventional CRT upgrading impacts ventricular functional mitral regurgitation in the PICM population and whether the presence of significant MR is associated with impaired outcomes.

## Materials and methods

### Study design

The UPGRADE study (NCT01970423) was an investigator-initiated prospective randomized controlled trial to assess the accuracy of an impedance sensor to detect sleep apnea and to evaluate the effects of conventional CRT upgrading in patients with central sleep apnea. Despite the fact that the randomized controlled design focused exclusively on patients with central sleep apnea, all other patients also received a thorough examination, including electrocardiography, transthoracic echocardiography, and laboratory tests, prior to and 3–5 months post-CRT activation in an observational fashion. The complete study design has been described in previous publications (13–15). Inclusion criteria were heart failure symptoms despite guideline-directed medical therapy (New York Heart Failure Association Class II, III, or ambulatory IV), an LVEF below 40%, and an ongoing need for RV pacing above 40% despite optimization of device programming. Exclusion criteria were end-stage heart failure defined by vasopressor dependency or low output syndrome, severe renal insufficiency with a glomerular filtration rate below 30 mL/min/1.73 m^2^, a remaining life expectancy below 1 year, women with childbearing potential, drug abuse, hyperthyroidism, or an intolerance to contrast agent. The primary endpoint of this *post-hoc* analysis was the assessment of MR improvement and change in MR grading 3–5 months post-CRT activation in patients with PICM. MR grading was based on echocardiography prior to and post-CRT activation in all patients and multiparametric assessment as proposed by American (ASE) and European (EACVI) experts (16, 17). CRT response was defined as a decrease in left ventricular end-systolic volume of more than 15%. Survival status was obtained either by telephone contact, the hospital information system, or during a recent ambulatory visit. Patients who were lost to follow-up were censored in the survival analysis at the last point of contact. Optimization of device programming, including atrioventricular (AV) and ventricular-ventricular (VV) interval, was kept unchanged during the initial phase of the study (including both echocardiographic examinations) until the main hypothesis of UPGRADE was concluded, but was able to be adapted during long-term follow-up at the physician's discretion (13–15). Each study patient provided verbal and written informed consent prior to enrolment. The study was conducted in accordance with the requirements of good clinical practice and the ethical principles of the Helsinki Declaration and approval from the local ethics committee (Medical University of Innsbruck) was obtained.

### Statistics

Categorical parameters are described as numbers with percentages, and continuous parameters are listed as median and interquartile range or 95% confidence interval (CI) whenever particularly indicated. The delta between paired variables (e.g., LVEF) was calculated by subtracting baseline measurements from measurements after therapy (e.g., LVEF post-CRT–baseline LVEF) and is presented as median and 95% CI. Distribution of continuous variables was assessed using the Shapiro–Wilk test and inspection of histograms and quantile-quantile plots. Mann–Whitney U or independent *t*-tests were used to analyze differences in independent variables, whereas the paired *t*-test or the Wilcoxon test was utilized in paired continuous parameters, each according to their distribution. Comparisons of categorical variables were performed by using Fisher’s exact test, and differences in paired ordinal variables were assessed using the McNemar–Bowker test. Bivariate correlations were calculated strictly using Spearman’s correlation coefficient since neither of the target variables was normally distributed. Differences in survival were illustrated using Kaplan–Meier curves, while statistical significance was assessed with the log-rank test. To further evaluate the influence of significant MR, defined as moderate or worse, a multivariable Cox regression model was calculated, adjusting for LVEF, calculated systolic pulmonary pressures, and N-terminal pro-brain natriuretic peptide (NT-proBNP) plasma levels. IBM SPSS, version 21 (IBM Corporation, Armonk, NY, USA) was used for the statistical analyses and the graphics were designed using GraphPad PRISM, version 5 (GraphPad Software, Inc., La Jolla, CA, USA). The whiskers in the presented box plot demonstrate the 95% confidence interval (2.5%–97.5%). *P*-values ≤ 0.05 were considered statistically significant.

## Results

### Study population and baseline characteristics

**Figure 1:**
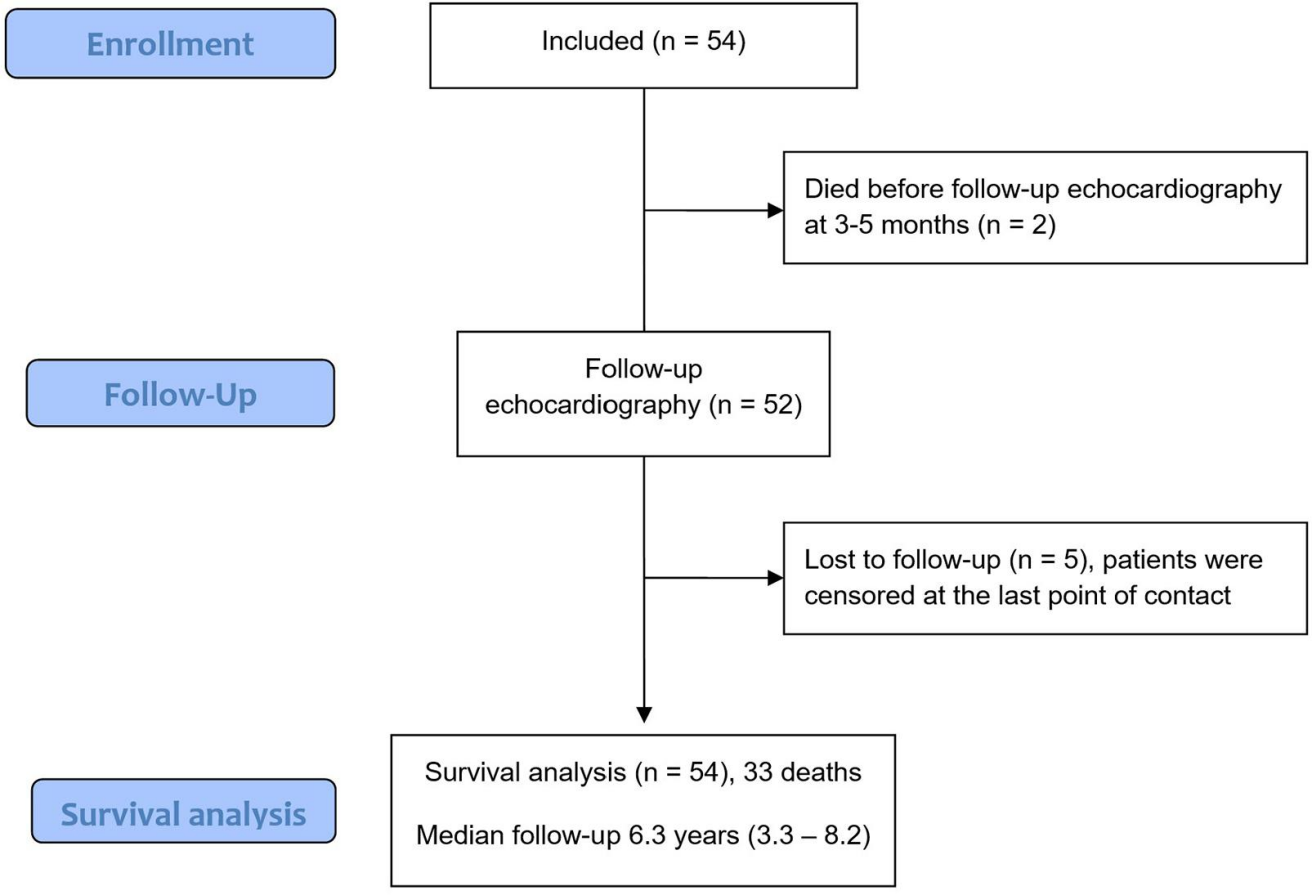
CONSORT diagram.

Overall, 54 patients were enrolled between 2014 and 2019. Two patients died prior to undergoing follow-up assessment of CRT effects. Five patients were lost to follow-up, whereas 33 (67.3%) probands died during long-term follow-up (Figure 1). Baseline characteristics are shown in Table 1. Importantly, the patients were 75.0 (71.5–78.0) years old and 33 (61.1%) had received a CRT pacemaker device. Given the inclusion criteria, all the patients had symptomatic heart failure, with the majority classified as New York Heart Association class III (*n* = 39, 72.2%). MR was predominantly found to be of ventricular functional etiology in nearly all cases (*n* = 52, 96.3%), while two cases (3.7%) were predominantly due to atrial dilatation, hence of atrial functional origin.

**Table 1:** Baseline characteristics.

Variable	Overall (*n* = 54)
Gender (male sex)	43 (79.6%)
Age (years)	75.0 (71.5–78.0)
BMI	26.2 (24.0–28.5)
CRT-P	33 (61.1%)
CABG	14 (25.9%)
PCI	12 (22.2%)
MVR	5 (9.3%)
TVR	5 (9.3%)
AVR	6 (11.1%)
AVN ablation	5 (9.3%)
COPD	11 (20.4%)
Atrial fibrillation	31 (57.5%)
New York Heart Association class	
II	12 (22.2%)
III	39 (72.2%)
IV	3 (5.6%)
Etiology of mitral regurgitation	
Atrial functional	2 (3.7%)
Ventricular functional	52 (96.3%)
Medication	
Betablocker	43 (79.6%)
Mineralocorticoid antagonist	29 (53.7%)
ACE inhibitor/ARB/ARNI	46 (85.2%)
Loop diuretic	47 (87.0%)
Hydrochlorothiazide	6 (11.1%)
Xipamid	5 (9.3%)
Response to cardiac resynchronization therapy	30 (55.6%)

Numbers are presented as median (interquartile range) or number of patients (percentage). ACE, angiotensin converting enzyme; ARB, angiotensin receptor blocker; ARNI, angiotensin receptor-neprilysin inhibitor; AVN, atrioventricular node; AVR, aortic valve replacement; BMI, body mass index; CABG, coronary artery bypass grafting; COPD, chronic obstructive pulmonary disease; CRT-P, cardiac resynchronization therapy pacemaker; MVR, mitral valve repair; PCI, percutaneous coronary intervention; TVR, tricuspid valve repair.

### The effect of conventional cardiac resynchronization therapy upgrading on functional mitral regurgitation

**Figure 2:**
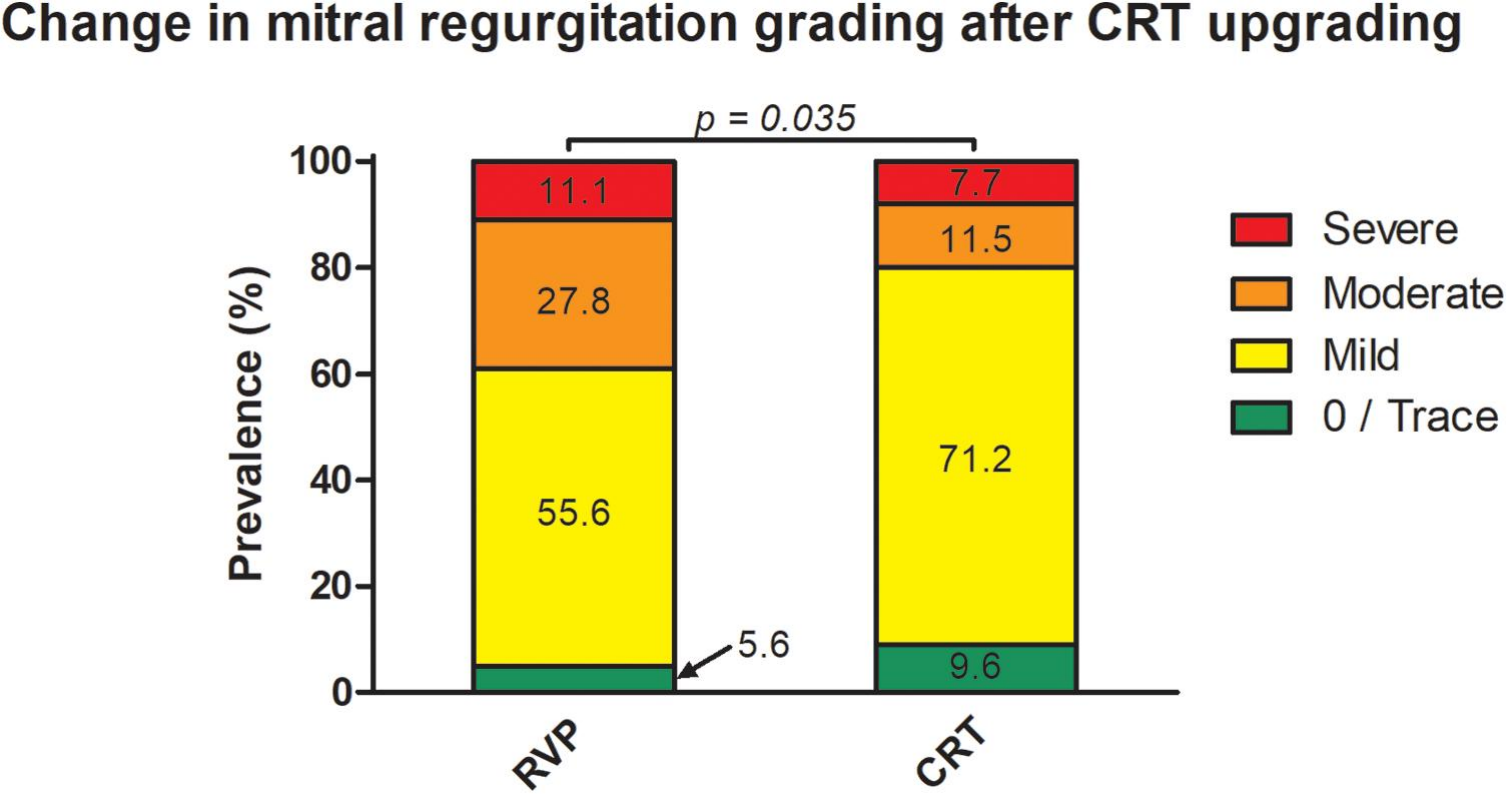
Change in MR grading after CRT upgrading.

**Figure 3:**
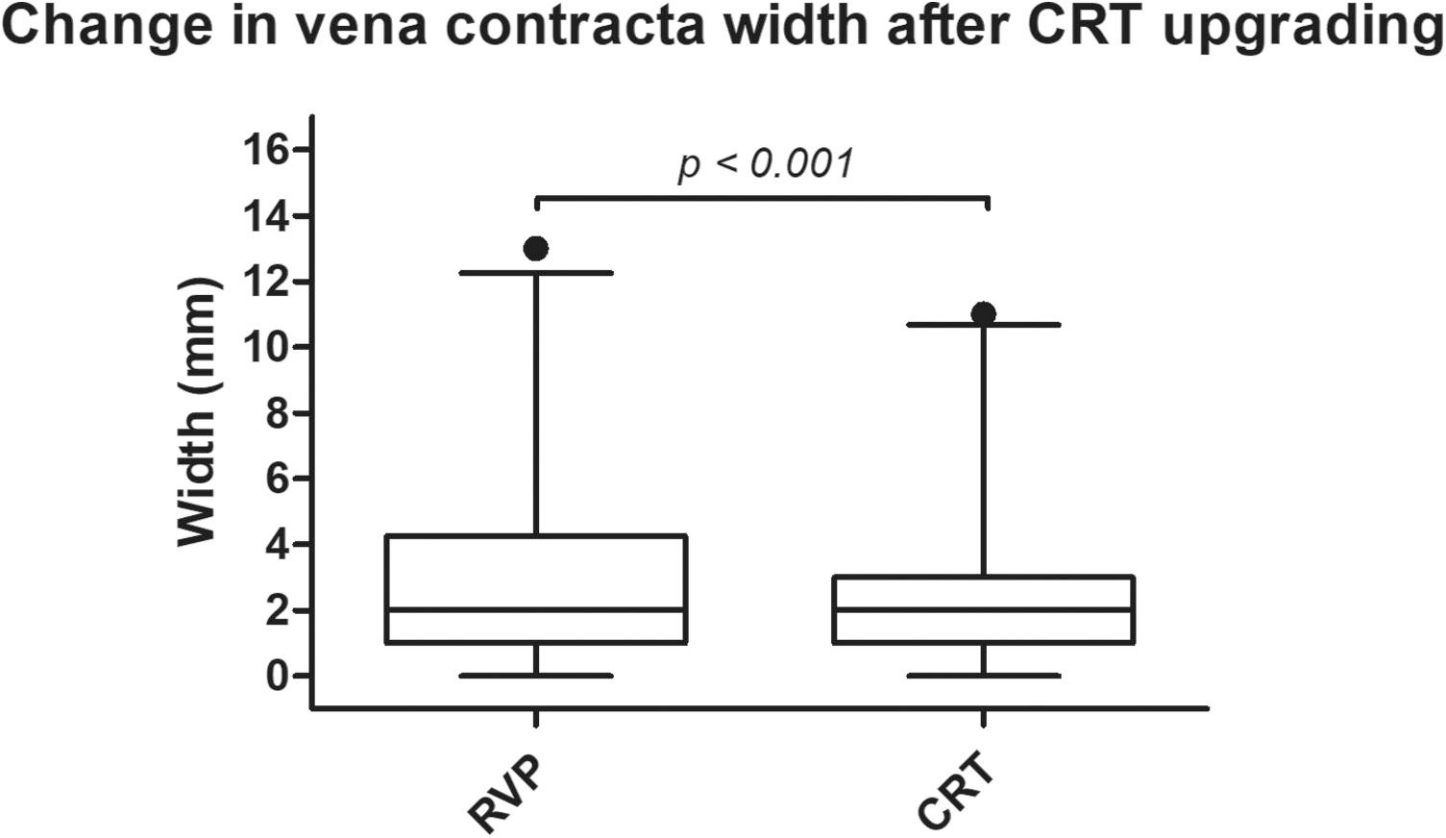
Change in VC width after CRT upgrading.

At baseline evaluation, the severity of MR was graded as “none/trace” in 3 (5.6%) patients, whereas the majority had mild MR (*n* = 30, 55.6%). Moderate MR was detected in 15 (27.8%) patients and severe MR was found in 6 (11.1%) patients. Accordingly, 21 (38.9%) probands were found to have at least moderate MR. Overall, a CRT response was observed in 30 (55.6%) patients. Upgrading to CRT was associated with a statistically significant reduction in MR [“none/trace”: *n* = 5 (9.6%), mild: *n* = 37 (71.2%), moderate: *n* = 6 (11.5%), severe: *n* = 4 (7.7%); *p* = 0.035]. An improvement in MR grading was found in 12 patients (22.2%), whereas the majority had no change in MR grading (Figure 2). Importantly, only two (33.3%) patients with severe MR at baseline improved to either mild (*n* = 1, 16.7%) or moderate MR (*n* = 1, 16.7%). Nine patients (60.0%) with moderate MR improved to either mild (*n* = 7, 46.7%) or “none/trace” MR (*n* = 2, 13.3%). Only one patient (3.3%) improved from mild to “none/trace” MR. Beyond overall classification, it was also linked to a significant reduction in vena contracta (VC) width [2 mm (1.0–4.3) at baseline versus 2 mm (1.0–3.0) post-CRT; *r* = 0.73, 95% CI 0.53–0.85, *p* < 0.001]. VC width was found to be either improved or steady in all but four (7.4%) patients at follow-up assessment (Figure 3). The effect of CRT upgrading on other parameters is shown in Table 2. Most importantly, it was linked to a reduction in QRS width [delta of −40 ms (95% CI −101 to −12)], left ventricular end-diastolic volumes [(LVEDV) delta of −11 mL (−128–73)], left ventricular end-systolic volumes [(LVESV) delta of −25 mL (−108–43)] and NT-proBNP plasma levels [delta of −636 ng/L (−19,970–3,251)], while improving LVEF [delta of 12% (−3–31)].

**Table 2:** Descriptive laboratory, electro-, and echocardiographic data under right ventricular pacing and cardiac resynchronization therapy.

Variable	RVP	CRT	delta (95% CI)
Laboratory tests			
NT-proBNP (ng/L)	2,354 (1,236–4,690)	1421 (756–2,879)	−636 (−19,970 to 3,251)
Creatinine (mg/dL)	1.37 (1.07–1.61)	1.37 (1.09–1.59)	0.03 (−0.47 to 0.45)
Echocardiography			
LVEF (%)	28.0 (21.9–35.9)	43.0 (33.0–52.0)	12 (−3 to 31)
LVEDV (mL)	182 (139–223)	163 (124–211)	−11 (−128 to 73)
LVESV (mL)	132 (97–160)	84 (62–138)	−25 (−108 to 43)
LVEDD (mm)	66 (56–71)	59 (54–66)	−3 (−41 to 12)
LVESD (mm)	54 (47–60)	47 (37–55)	−6 (−25 to 16)
TAPSE (mm)	18 (15–22)	19 (16–23)	0 (−9 to 15)
LA volume A4C (mL)	143 (99–179)	122 (94–155)	−1 (−134 to 63)
LA volume A2C (mL)	147 (107–216)	144 (97–185)	−6 (−124 to 77)
Calculated sPAP (mmHg)	35 (29–43)	33 (26–40)	−3 (−28 to 20)
Electrocardiogram			
QRS (ms)	195 (176–209)	148 (134–166)	−40 (−101 to −12)

Numbers are presented as median (interquartile range, or 95% confidence interval if indicated) or number of patients (percentage). A2C, apical two-chamber view; A4C, apical four-chamber view; CI, confidence interval; CRT, cardiac resynchronization therapy; LA, left atrial; LVEDD, left ventricular end-diastolic diameter; LVEDV, left ventricular end-diastolic volume; LVEF, left ventricular ejection fraction; LVESD, left ventricular end-systolic diameter; LVESV, left ventricular end-systolic volume; NT-proBNP, N-terminal pro-brain natriuretic peptide; RVP, right ventricular pacing; sPAP, systolic pulmonary artery pressure; TAPSE, tricuspid annular plane systolic excursion.

### Functional mitral regurgitation and its effect on long-term outcomes in patients undergoing cardiac resynchronization therapy upgrading

**Figure 4:**
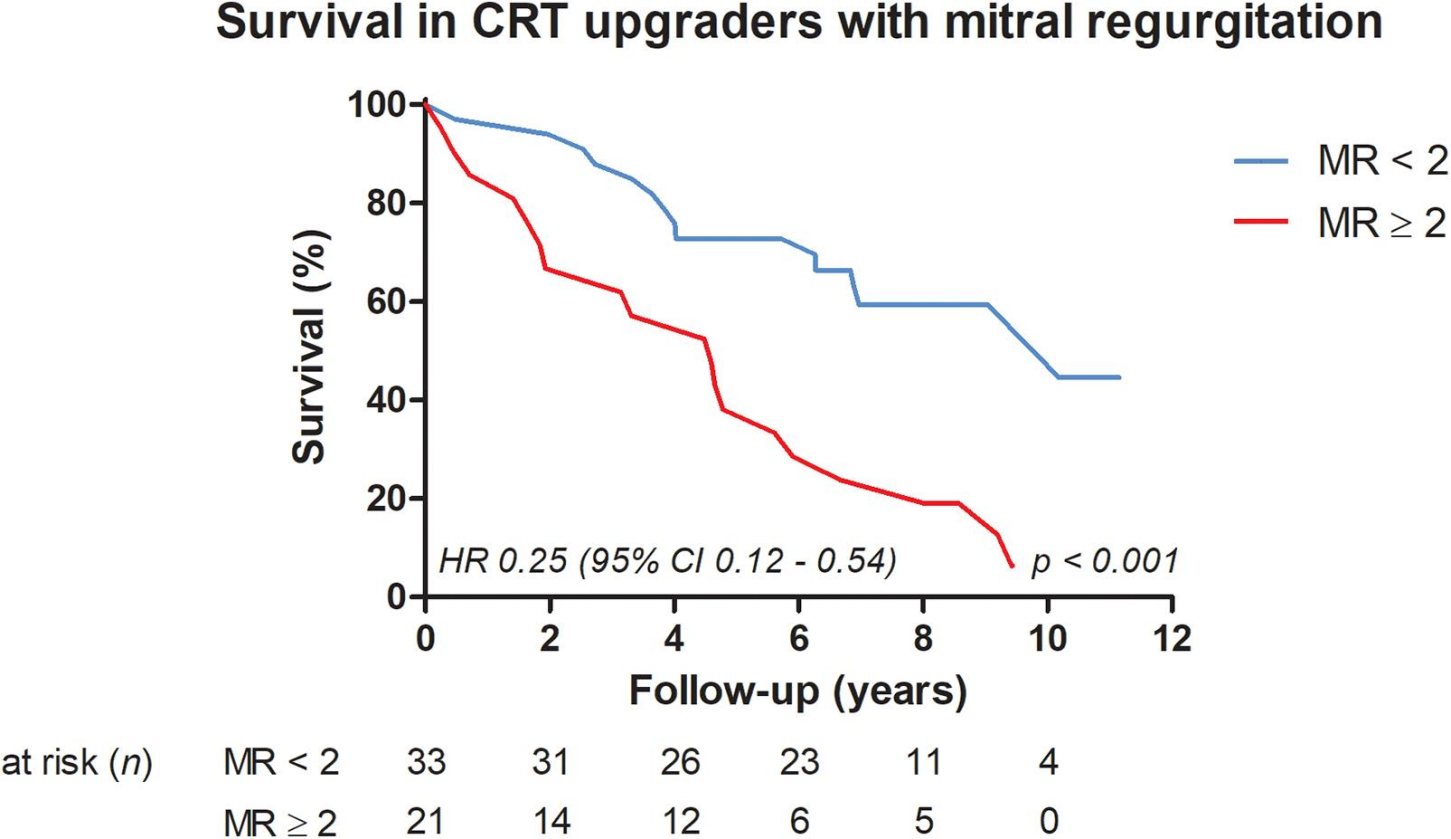
Survival in CRT upgraders with MR.

Patients with moderate or severe MR at baseline (MR ≥ 2) were associated with impaired long-term outcomes [follow-up of 6.3 years (3.3–8.2)], yielding statistically significant higher all-cause mortality [MR < 2: *n* = 14 (42.4%), MR ≥ 2: *n* = 19 (90.5%); hazard ratio 0.25 (95% CI 0.12–0.54); log rank *p* < 0.001, Figure 4]. These patients were associated with statistically significantly more dilated left atrial volumes in the apical four-chamber [164 mL (120–200) versus 117 mL (92–175); *p* = 0.036] and apical two-chamber views [162 mL (123–232) versus 127 mL (93–185); *p* = 0.020) and an increased calculated systolic pulmonary artery pressures [40 mmHg (35–50) versus 31 mmHg (27–41); *p* = 0.010]. There was also a tendency for higher NT-proBNP plasma levels [3327 ng/L (1,848–6,375) versus 1805 ng/l (1,119–3,881); *p* = 0.064] and lower LVEF [27.0% (21.4–33.5) versus 32.0% (22.0–38.5); *p* = 0.094], although neither reached the level of statistical significance (Table 3). Even after adjustment for LVEF, calculated systolic pulmonary artery pressures, and NT-proBNP plasma levels, moderate or severe MR at baseline remained a strong predictor of higher all-cause mortality [hazard ratio 0.38 (95% CI 0.18–0.81); *p* = 0.012].

**Table 3:** Comparison of laboratory, electro-, and echocardiographic data in patients with moderate mitral regurgitation or greater to patients with less than moderate mitral regurgitation at baseline.

Variable	MR ≥ 2	MR < 2	*p*-value	Cohen's *D*/r*	95% CI
Laboratory tests					
NT-proBNP (ng/L)	3,327 (1,848–6,375)	1,805 (1,119–3,881)	0.064	−0.252*	−0.496 to 0.011
Creatinine (mg/dL)	1.35 (1.05–1.66)	1.38 (1.09–1.60)	0.498	−0.191	−0.774 to 0.390
Echocardiography					
LVEF (%)	27.0 (21.4–33.5)	32.0 (22.0–38.5)	0.094	0.476	−0.070 to 1.079
LVEDV (mL)	172 (145–212)	186 (138–231)	0.434	0.22	−0.281 to 0.721
LVESV (mL)	141 (95–158)	122 (95–161)	0.859	−0.024*	−0.303 to 0.240
LVEDD (mm)	67 (58–73)	62 (56–71)	0.49	−0.096*	−0.356 to 0.178
LVESD (mm)	55 (47–61)	53 (47–57)	0.94	0.021	−0.594 to 0.546
TAPSE (mm)	17 (14–21)	18 (16–22)	0.159	0.399	−0.127 to 0.967
LA volume A4C (mL)	164 (120–200)	117 (92–175)	0.036	−0.648	−1.333 to −0.038
LA volume A2C (mL)	162 (123–232)	127 (93–185)	0.02	−0.722	−1.389 to −0.152
Calculated sPAP (mmHg)	40 (35–50)	31 (27–41)	0.01	−0.762	−1.487 to −0.210
Electrocardiogram					
RVP QRS (ms)	195 (176–209)	192 (174–205)	0.276	−0.308	−0.868 to 0.213

Numbers are presented as median (interquartile range) or number of patients (percentage). A2C, apical two-chamber view; A4C, apical four-chamber view; CI, confidence interval; LA, left atrial; LVEDD, left ventricular end-diastolic diameter; LVEDV, left ventricular end-diastolic volume; LVEF, left ventricular ejection fraction; LVESD, left ventricular end-systolic diameter; LVESV, left ventricular end-systolic volume; NT-proBNP, N-terminal pro-brain natriuretic peptide; RVP, right ventricular pacing; sPAP, systolic pulmonary artery pressure; TAPSE, tricuspid annular plane systolic excursion. Asterisks mark non-parametric variables with respective calculation of effect size r; all others display Cohen's D.

### Predictors of improvement in functional mitral regurgitation due to cardiac resynchronization therapy upgrading

To assess predictors of improvement in functional MR due to CRT upgrading, several parameters were compared between patients with MR improvement and patients without. None of following demonstrated statistically significant differences: the delta of NT-proBNP (*p* = 0.481), LVEF (*p* = 0.768), LVEDV (*p* = 0.169), LVESV (*p* = 0.105), left ventricular end-diastolic diameter (*p* = 0.382), left ventricular end-systolic diameter (*p* = 0.256), tricuspid annular plane systolic excursion (*p* = 0.763), left atrial volume (apical four-chamber view, *p* = 0.330; apical two-chamber view, *p* = 0.556), calculated systolic pulmonary artery pressure (*p* = 0.208), and QRS width (*p* = 0.833). Furthermore, both CRT response (*p* = 0.318) and a known history of atrial fibrillation failed to predict an improvement (*p* = 1.000).

## Discussion

The present analysis has revealed several important new findings. First, functional MR is highly prevalent in patients with PICM undergoing CRT upgrading, with approximately 40% suffering from at least moderate MR. Second, this is the first analysis to demonstrate an improvement in functional MR in approximately 25% of patients who underwent conventional CRT upgrading. Third, moderate or severe MR was associated with impaired survival in this cohort of CRT upgraders, with mortality rates approaching 90% during long-term follow-up, indicating that these patients represent a high-risk population. Moreover, MR was linked to more adverse atrial remodeling and increased pulmonary artery pressures, most likely reflecting postcapillary pulmonary hypertension, which itself is associated with worse prognosis (18).

Since MR appears to contribute to worse prognosis in patients with PICM, we aimed to evaluate the potential effect of CRT upgrading in managing MR, showing a statistically significant association with improvement in MR. However, the effect on severe mitral regurgitation seemed rather modest as the majority (*n* = 4, 66.7%) had the same grading at follow-up, highlighting another important clinical aspect. While the following therapeutic considerations are not derived from the present data, the findings raise several clinical hypotheses for future investigation, as these patients may benefit from subsequent prognostic therapies such as mitral transcatheter edge-to-edge repair. It remains nonetheless unclear how long to wait for a potential therapeutic response before proceeding with such adjunct therapies, especially in the light of high mortality rates (19, 20). Given the limited evidence and availability of mitral valve interventions at the time of this study, there was no influence due to MR amelioration through other invasive valve therapies on the prognosis assessment, which can be seen as another strength of the presented study. A previous trial assessing the effect of *de novo* implantation of conventional CRT on moderate to severe functional MR similarly reported a high mortality rate of 13% before re-assessment at a 6-month post-implantation follow-up was even conducted. The rate of MR improvement in *de novo* implantations (∼50%) was also higher compared to that observed (∼25%) in our CRT upgrading cohort, whereas MR improvement was associated with significantly improved survival (12). This difference may be partly due to the heterogeneity of patients who meet the definition of PICM. Some of them may also experience heart failure progression irrespective of RV pacing and are not expected to respond to a similar extent, but rather be stabilized. To counterbalance this, a stricter definition (RV pacing of 40% or above) was used as an inclusion criterion in the UPGRADE study. Ultimately, the high prevalence of atrial fibrillation (57.5%) may also have influenced this limited improvement in MR, as was found to reduce the response to CRT upgrading in previous trials (21, 22).

Several mechanisms of MR reduction in CRT recipients have been reported. On the one hand, there is an optimization of the force–balance relationship caused by reduced tethering and improvement in closing forces. The reduced tethering of the leaflets itself is mainly driven by improved left ventricular geometrics due to a reversal of structural remodeling, which is typically observed over time (23). On the other hand, an acute effect due to the resynchronization of the papillary muscles has been described. Ypenburg et al. investigated the effect of CRT on the dyssynchrony of the anterior and posterior papillary muscles, showcasing a tremendous electrical improvement (interpapaillary delay of 169 ± 69 ms reduced to 25 ± 26 ms), which was associated with a reduction in MR severity directly after CRT activation (24). Given this multifactorial nature and since no differentiation between acute and long-term effects was made, our exploratory analysis failed to demonstrate any specific significant predictor of MR improvement.

The general principle behind CRT, i.e., to aim for optimal resynchronization, should also be acknowledged to ameliorate functional MR. The factors shown to be clearly associated with reversal of structural remodeling are QRS width reduction and QRS area/vector normalization (25–27). Whenever conventional CRT is not sufficient, the following further strategies can be implemented to achieve/improve the resynchronization:

- multipoint pacing with dedicated leads (28),
- multisite pacing and combined strategies such as HOT-CRT or LOT-CRT (29, 30), and
- AV and VV interval optimization, especially in patients with sinus rhythm and preserved atrioventricular conduction. Although historically conducted by echocardiography, newer algorithms such as SonR (Microport CRM, Clarmart, France), Adaptive CRT (Medtronic, Dublin, Ireland), and SyncAV (Abbott Laboratories, Abbott Park, IL, USA) allow for automated, continuously performed optimization (31–33).

It remains to be seen whether conduction system pacing will replace conventional CRT in the future since electrical activation of the ventricles seems to be further improved (34, 35). To the best of our knowledge, there is no distinct analysis of the effects of any form of conduction system pacing on MR grading. There is only one very small trial proclaiming beneficial effects as a secondary endpoint (36). Given the prescribed mechanisms for MR improvement and the improved electrical activation by conduction system pacing, it is conceivable that conduction system pacing could achieve a more significant improvement in functional MR. Importantly, CRT upgrading is still one of the driving indications for CRT overall, with approximately one-quarter of patients having pre-existing cardiac implantable electronic devices, further underlining the necessity of research on this topic (37).

There are several limitations that have to be acknowledged. The presented study included only a small study cohort, although it was similar in size to previous trials investigating the effect of CRT on MR in *de novo* implantations. Furthermore, it was conducted as a *post-hoc* analysis with all adherent potential forms of bias and has to be viewed as a hypothesis-generating exploratory analysis. Although nearly all the cases of MR were found to be of predominantly ventricular functional origin, a potential minor contribution of atrial dilatation, as found in atrial functional MR, cannot be excluded. The percentage of biventricular pacing and left ventricular lead positioning, as well as their potential influence on MR improvement, were not evaluated in this study. Given the prespecified timepoint for echocardiographic follow-up assessment of 3–5 months post-CRT upgrading, a small proportion of late responders may have been missed, which is thought to be possible until 12 months post-implant. The absence of a core lab or dual-reader protocol with all adherent forms of bias is acknowledged. Correction for simultaneous testing, as conducted in Table 3, was not conducted due to the small study cohort. Informative censoring among the five patients who were lost to follow-up cannot be excluded. A particular strength of this study was the availability of long-term follow-up.

## Conclusions

Conventional cardiac resynchronization therapy upgrading in patients with PICM was associated with significant improvement of functional MR in terms of grading and vena contracta width, yet was observed in only approximately 25% of patients. The presence of significant MR was linked to impaired long-term survival and more pronounced atrial remodeling, emphasizing the need for close monitoring of these patients to provide timely additional therapies such as mitral transcatheter edge-to-edge repair.

## Data Availability

The raw data supporting the conclusions of this article will be made available by the authors, without undue reservation.
